# High-quality brain perfusion SPECT images may be achieved with a high-speed recording using 360° CZT camera

**DOI:** 10.1186/s40658-020-00334-7

**Published:** 2020-11-04

**Authors:** Manon Bordonne, Mohammad B. Chawki, Pierre-Yves Marie, Timothée Zaragori, Véronique Roch, Rachel Grignon, Laetitia Imbert, Antoine Verger

**Affiliations:** 1 Department of Nuclear Medicine and Nancyclotep Imaging Platform, CHRU-Nancy, Université de Lorraine, F-54000 Nancy, France; 2 Médecine Nucléaire, CHRU-Nancy Brabois, Allée du Morvan, 54500 Vandoeuvre-lès-, Nancy, France; 3 Université de Lorraine, INSERM, UMR-1116 DCAC, F-54000 Nancy, France; 4 Université de Lorraine, INSERM U1254, IADI, F-54000 Nancy, France

**Keywords:** CZT, Brain perfusion, SPECT, Image quality, Count sensitivity

## Abstract

**Objective:**

The aim of this study was to compare brain perfusion SPECT obtained from a 360° CZT and a conventional Anger camera.

**Methods:**

The 360° CZT camera utilizing a brain configuration, with 12 detectors surrounding the head, was compared to a 2-head Anger camera for count sensitivity and image quality on 30-min SPECT recordings from a brain phantom and from ^99m^Tc-HMPAO brain perfusion in 2 groups of 21 patients investigated with the CZT and Anger cameras, respectively. Image reconstruction was adjusted according to image contrast for each camera.

**Results:**

The CZT camera provided more than 2-fold increase in count sensitivity, as compared with the Anger camera, as well as (1) lower sharpness indexes, giving evidence of higher spatial resolution, for both peripheral/central brain structures, with respective median values of 5.2%/3.7% versus 2.4%/1.9% for CZT and Anger camera respectively in patients (*p* < 0.01), and 8.0%/6.9% versus 6.2%/3.7% on phantom; and (2) higher gray/white matter contrast on peripheral/central structures, with respective ratio median values of 1.56/1.35 versus 1.11/1.20 for CZT and Anger camera respectively in patients (*p* < 0.05), and 2.57/2.17 versus 1.40/1.12 on phantom; and (3) no change in noise level. Image quality, scored visually by experienced physicians, was also significantly higher on CZT than on the Anger camera (+ 80%, *p* < 0.01), and all these results were unchanged on the CZT images obtained with only a 15 min recording time.

**Conclusion:**

The 360° CZT camera provides brain perfusion images of much higher quality than a conventional Anger camera, even with high-speed recordings, thus demonstrating the potential for repositioning brain perfusion SPECT to the forefront of brain imaging.

**Supplementary Information:**

The online version contains supplementary material available at 10.1186/s40658-020-00334-7.

## Key points

Question: how does the 360° CZT camera utilizing brain configuration mode contribute to the enhancement of brain perfusion SPECT investigations undertaken in clinical routine?

Pertinent findings: compared with the Anger camera, the CZT camera provides greater than 2-fold increase in count sensitivity and higher image quality performance (up to 119% of enhancement in spatial resolution and 41% in gray/white matter contrast). These image quality enhancements are observed both quantitatively and visually, with all performance parameter results remaining unchanged even when CZT images are obtained with reduced 15 min recording time.

Implications for patient care: high-quality brain perfusion SPECT images may be achieved with high-speed recording using a 360° CZT camera, thus being particularly useful in neurological disease patients for whom a brain perfusion SPECT may be indicated.

## Introduction

Brain perfusion SPECT is considered a useful investigation to estimate regional blood flow and is recommended [1] as part of the full diagnostic workup for various indications—i.e., dementia, cerebrovascular diseases, before surgery for epilepsy, traumatic injuries, inflammatory diseases, and brain death [2–9]. To date however, brain perfusion SPECT has suffered from sub-optimal image quality with limited spatial resolution [10].

In more recent years, owing to developments in gamma camera technology with the introduction of cadmium-zinc-telluride (CZT) semiconductors and original collimation systems, marked improvements in SPECT image quality have been clearly established [11–16], mainly due to the significant impact of such technological advances on both intrinsic spatial [17] and energy resolution [18, 19]. Moreover, certain CZT cameras equipped with original collimation systems provide higher tomographic count sensitivities, thus allowing reductions in recording time and/or injected activity [20]. In particular, a 360° CZT camera (Veriton®, Spectrum Dynamics Medical) combines high performance CZT detectors with an original 360° ring configuration geometry that allows the detectors to be positioned very close to the patient’s head [20], thus providing a configuration for brain imaging that is likely to enhance both count sensitivity and image quality. Nonetheless, most prior studies comparing CZT with Anger cameras have been conducted in the context of myocardial perfusion imaging [21–28], and very limited data is available for brain perfusion SPECT [18]. It is therefore considered to what extent does this new 360° CZT camera using brain configuration mode contribute to the enhancement of brain perfusion SPECT investigations undertaken in clinical routine.

The aim of this study is to quantitatively and qualitatively evaluate the quality of brain perfusion SPECT images from both phantom and patients, obtained using the 360° CZT camera in dedicated brain configuration mode, and to make comparison with Anger camera images.

## Material and methods

### Study population

A total of 21 patients, who underwent ^99m^Tc-HMPAO brain SPECT on the 360° CZT camera (Veriton, Spectrum Dynamics Medical®) in our department of Nuclear Medicine from October 2018 to March 2019, were retrospectively included. They were matched according to gender and age (± 7 years) to 21 other patients for whom this investigation had been performed earlier with an Anger camera (Symbia T2, Siemens Healthineers®) during the period February 2013 to March 2018. All patients within these 2 groups were included only if they met the following criteria: greater than 18 years old; without dementia according to the Diagnostic and Statistical Manual of Mental Disorders 5th Ed. (DSM V, [29]) and with a Mini-Mental State Examination (MMSE) at least equal to 15; and without any brain SPECT/CT abnormality at visual analysis.

All procedures performed in this study involving human participants were in accordance with the ethical standards of the institutional research committee and with the principles of the 1964 Declaration of Helsinki and its later amendments or comparable ethical standards. The study was approved on July 1, 2019, by the Ethics Committee of the CHRU of Nancy (reference number 238).

### Patients’ SPECT/CT recordings

SPECT/CT recordings on both cameras commenced after the injection of 684.5 ± 80.0 MBq of ^99m^Tc-HMPAO (corresponding to a mean effective dose of 6.4 mSv) and a subsequent 15 min period of neurosensory rest in a dedicated room which limited auditory and visual stimulation.

The CT scan was performed first using the following parameters: 110 kV, 70 mAs, pitch of 1.0, and slice thickness of 3 mm for the Anger camera; and 120 kV, 54 mAs, pitch of 0.75, and slice thickness of 2.5 mm for the CZT camera, leading to an effective dose of approximately 0.4 mSv for both cameras.

SPECT recording was planned with a 30 min acquisition period, over 360° and with an energy window of 140 keV ± 7.5% for both cameras. More precisely, for each of the 2 heads of the Anger camera which were placed at 180° apart and equipped with low-energy high-resolution (LEHR) parallel collimators, 32 frames were recorded through a 180° circular orbit with a radius of approximately 15.5 cm. For the CZT camera, as already detailed elsewhere [20], 12 high-resolution swiveling detectors were positioned close to the head around a 360° configuration, and a total of 2160 projections were recorded, of which 80% were focused on the parenchymal brain volume. This “focus mode” requires a short pre-scan recording of approximately 20 s to position the specific brain region of interest.

### Phantom experiment

A Hoffman 3-D Brain Phantom™ (Data Spectrum Corporation) was filled-in with a homogeneous solution of 96 MBq of ^99m^Tc to simulate the grey/white matter distribution of brain perfusion with a 4:1 uptake ratio (corresponding to the true contrast). This phantom was placed at the center of the field-of-view for both Anger and CZT cameras, and the SPECT/CT recording protocols with 30-min acquisition time were close to those used in patients, as already described above.

### Reconstruction of SPECT images

**Fig. 1:**
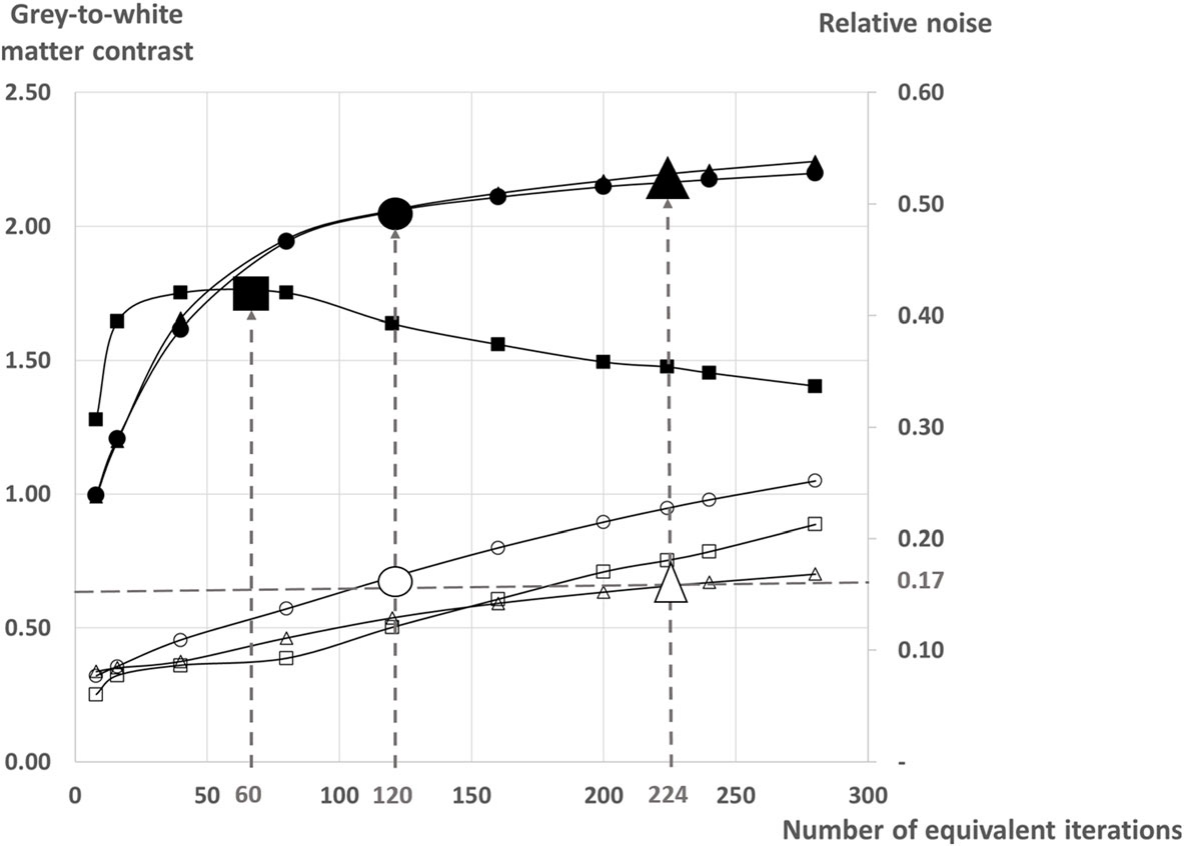
Evolution, according to the number of equivalent iterations of image reconstructions, of the grey-to-white matter contrast from occipital cortex (black symbols, corresponding scale on the left side) and of the relative noise index from semi-oval centers (white symbols, corresponding scale on the right side), on the SPECT images of the brain phantom recorded with the conventional Anger gamma camera (squares), as well as with full-time (triangles) and half-time (circles) recordings from the CZT camera. The number of equivalent iterations considered to reach a high enough convergence for contrast and with an acceptable noise level is indicated by vertical dashed lines. The level of relative noise reached by the CZT-camera at these optimal convergence levels (0.17 for both half-time and full-time recordings) is additionally indicated by a horizontal dashed line

The SPECT images from both cameras were reconstructed using an iterative OSEM reconstruction method, with corrections for attenuation and scattering and no additional filtering. Additionally, images provided by half-time recordings were simulated for the CZT camera, by suppression on the list-mode data of the counts recorded during the 2nd half of the recording time for each projection. And further, the iterative reconstruction parameters were selected according to the convergence for the grey/white matter contrast of the occipital area from the brain phantom. For this purpose, the number of recorded counts from the phantom was fixed at levels corresponding to those documented in clinical routine (on average, 8.8 and 16.9 million of total recording counts for the Anger and CZT cameras, respectively). In this way and as detailed in Fig. 1, the respective numbers of iterations and subsets were fixed at 4 and 15 for the Anger camera, and at 28 and 8 for the full-time 30 min CZT camera recordings. For the simulated half-time CZT camera recordings, the numbers of iterations and subsets were fixed at 15 and 8, thereby providing not only comparable levels for contrast but also an acceptable level of noise for clinical routine interpretation (< 0.20), as compared with the full-time 30 min recording.

Finally, brain SPECT images were displayed through cubic voxels of 3.9 × 3.9 × 3.9 mm^3^ for the Anger camera and of 2.46 × 2.46 × 2.46 mm^3^ for the CZT camera, and with a reorientation through the anterior-posterior commissure line.

### Performance parameters

*Tomographic count sensitivity*, expressed in counts.s^−1^.MBq^−1^, was calculated as the total recording counts divided by the acquisition time and by the injected activity corrected for decay at the recording time.

*Grey/white matter contrast* was determined for peripheral (occipital cortex) and central (striata) grey matter structures, as compared with the white matter semi-oval center, with the software LifeX® [30] and a previously described method where mean voxel counts (VC) are determined within 1 cm^3^ volumes of interest [31] and with the formula:

$$ \frac{\mathrm{VC}\ \left(\mathrm{grey}\ \mathrm{matter}\right)-\mathrm{VC}\ \left(\mathrm{white}\ \mathrm{matter}\right)}{\mathrm{VC}\ \left(\mathrm{white}\ \mathrm{matter}\right)} $$

*Spatial resolution* was assessed through a sharpness index measured at the grey/white matter interface of the aforementioned grey matter structure (occipital cortex and striata). As already described [31], this index is expressed in percent of the maximal count values and per millimeter length, and it is computed as the maximal slope of count variations through transverse count profiles generated perpendicularly to the grey/white matter interfaces, by using an open source software (ImageJ®, National Institutes of Health).

*Relative noise* was computed as the standard deviation to mean value ratio of voxel counts recorded in 1 cm^3^ volumes of interest in a homogeneous area of the white matter semi-oval center.

*Quality of brain perfusion images* was additionally scored visually with a 10-point graduation scale (from 1 for the lowest to 10 for the highest quality) by two blinded experienced observers (MB, AV) for the patients’ SPECT images from both cameras, presented in a random order.

### Statistical analysis

Median values with interquartile ranges were used for continuous variables (due to the non-normal distribution of some of them), and effectives and percentages were used for discrete variables. Two-group comparisons were planned with Chi-squared tests for discrete variables and with Mann-Whitney tests for continuous variables. In addition, Kruskal-Wallis tests with Bonferroni corrections were planned for the 3 group comparisons of continuous variables. For all tests, the statistical level of significance was set at *p* < 0.05. All statistical analyses were done with the SPSS 25.0 software (IBM®).

## Results

As detailed in Table 1, patients imaged with the CZT camera had equivalent characteristics to those imaged with the Anger camera, except for slightly but significantly lower values at MMSE test (*p* = 0.01).

**Table 1:** Main patient characteristics in the 2 groups of patients imaged with the Anger or CZT cameras

	Anger-camera group (*n* = 21)	CZT-camera group (*n* = 21)	*p* value
**Age (years)**	78 [70–81]	75 [73–80]	1.00
**Female gender**	12 (57%)	12 (57%)	1.00
**BMI (kg/m**^**2**^**)**	26.1 [24.1–28.7]	26.8 [23.2–28.6]	0.89
**MMSE** (/30)	27 [26–29]	24 [20–27]	0.01*
**Education**			0.90
0 = primary school	10 (48%)	9 (43%)	
1 = secondary school	4 (19%)	6 (29%)	
2 = high school	3 (14%)	3 (14%)	
3 = higher education	4 (19%)	3 (14%)	

*BMI* body mass index, *MMSE* Mini Mental State Examination test

*p < 0.05 between patients from Anger and SPECT cameras

Tomographic count sensitivity was a little more than 2-fold higher with the CZT camera than with the Anger camera, on both phantom (respectively 105.90 vs. 48.61 counts.s^−1^.MBq^−1^) and patients’ recordings (14.38 counts.s^−1^.MBq^−1^ vs. 6.79 counts.s^−1^.MBq^−1^, *p* < 0.001).

**Fig. 2:**
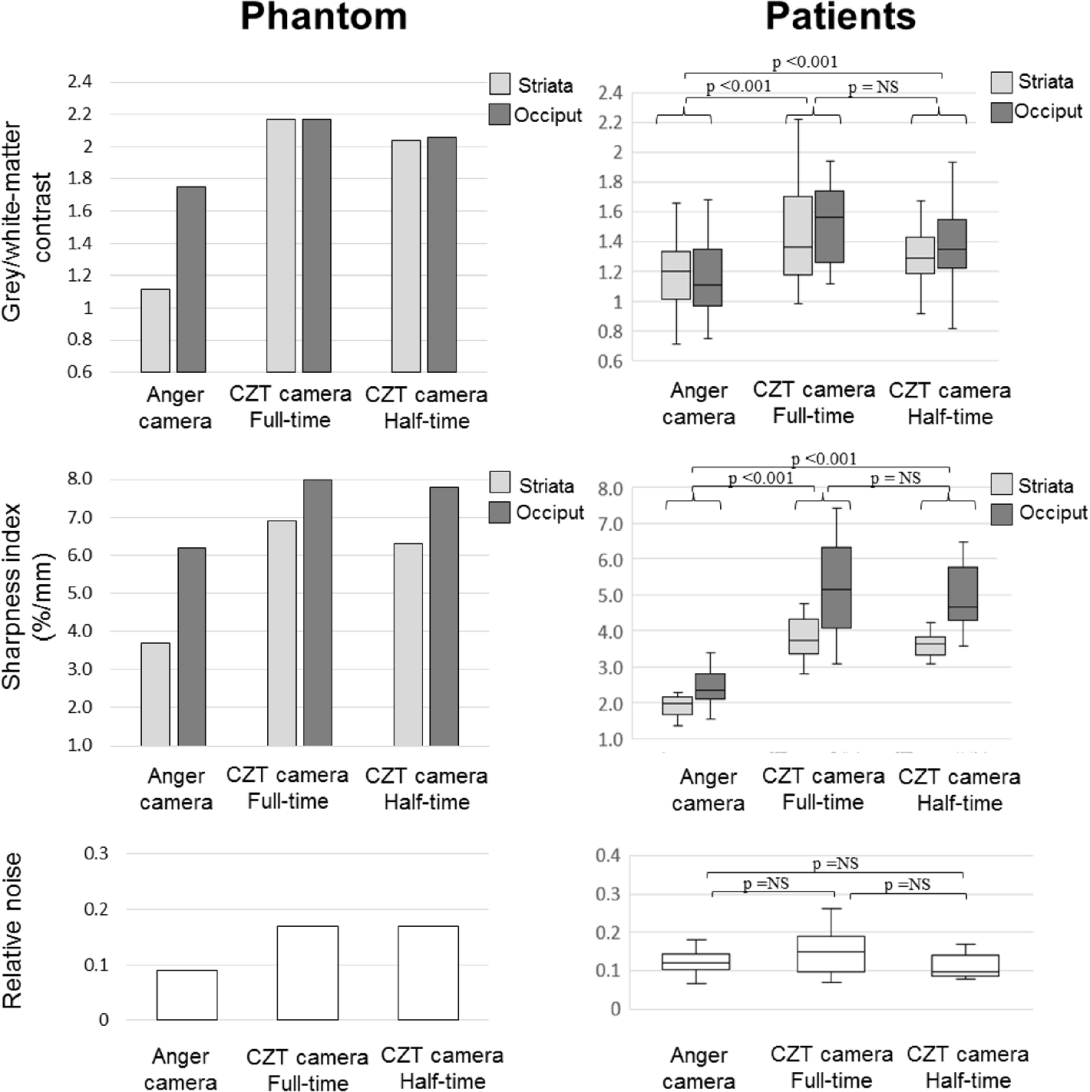
Grey/white matter contrast (upper panel), spatial resolution assessed through a sharpness index (middle panel), and relative noise (lower panel), obtained for peripheral (occipital cortex, dark grey columns), central grey matter (striata, light grey columns), and white matter structures (semi-oval centers, white columns) on the SPECT images of phantom (left panel) and patients (box-plots, right panel) with conventional Anger camera (left columns) and with full-time (median columns) and half-time (right columns) recordings from the CZT camera. *p* values significant in clinical study are indicated on the figure

As detailed in Fig. 2, most image quality parameters were also markedly enhanced with the CZT camera, as compared to the Anger camera, especially: (1) the spatial resolution assessed through a sharpness index for both peripheral and central brain structures, with respective median values 5.2% [interquartile range 4.2–6.2] and 3.7% [3.4–4.2] for CZT camera versus 2.4% [2.1–2.8] and 1.9% [1.7–2.2] for Anger camera in patients (all *p* < 0.01), and 8.0% and 6.9% for CZT camera versus 6.3% and 3.7% for Anger camera on the phantom; and (2) higher grey/white matter contrast on peripheral and central structures, with respective ratio median values of 1.56 [1.26–1.74] and 1.35 [1.19–1.65] for CZT camera versus 1.11 [0.97–1.33] and 1.20 [1.02–1.33] for Anger camera in patients (all *p* < 0.05), and 2.57 and 2.17 for CZT camera versus 1.40 and 1.12 for Anger camera on the phantom. By contrast, no significant change was documented between the 2 cameras for the relative noise level (*p* > 0.06, Fig. 2).

Furthermore, as evidenced on the data displayed in Fig. 2, these image quality parameters were not significantly changed on the CZT images obtained with a simulated half-time recording of only 15 min.

These results, obtained through objective quantitative parameters, could be supplemented by additional data from the visual analysis of experienced physicians. With visual analysis, a median value of as much as 80% was reached for the relative enhancement in quality score between the CZT and Anger cameras, suggesting that this enhancement was clear enough to be easily detected in clinical routine. More precisely, the median of the quality score was of 2.5 for Anger SPECT [interquartile range 2.0–3.0], and it was significantly higher for the CZT-SPECT images (*p* = 0.01), without any significant difference between images obtained with full-time (4.5 [3.0–5.0]) or simulated half-time (3.0 [2.0–3.5]) recordings (*p* = 0.17).

**Fig. 3:**
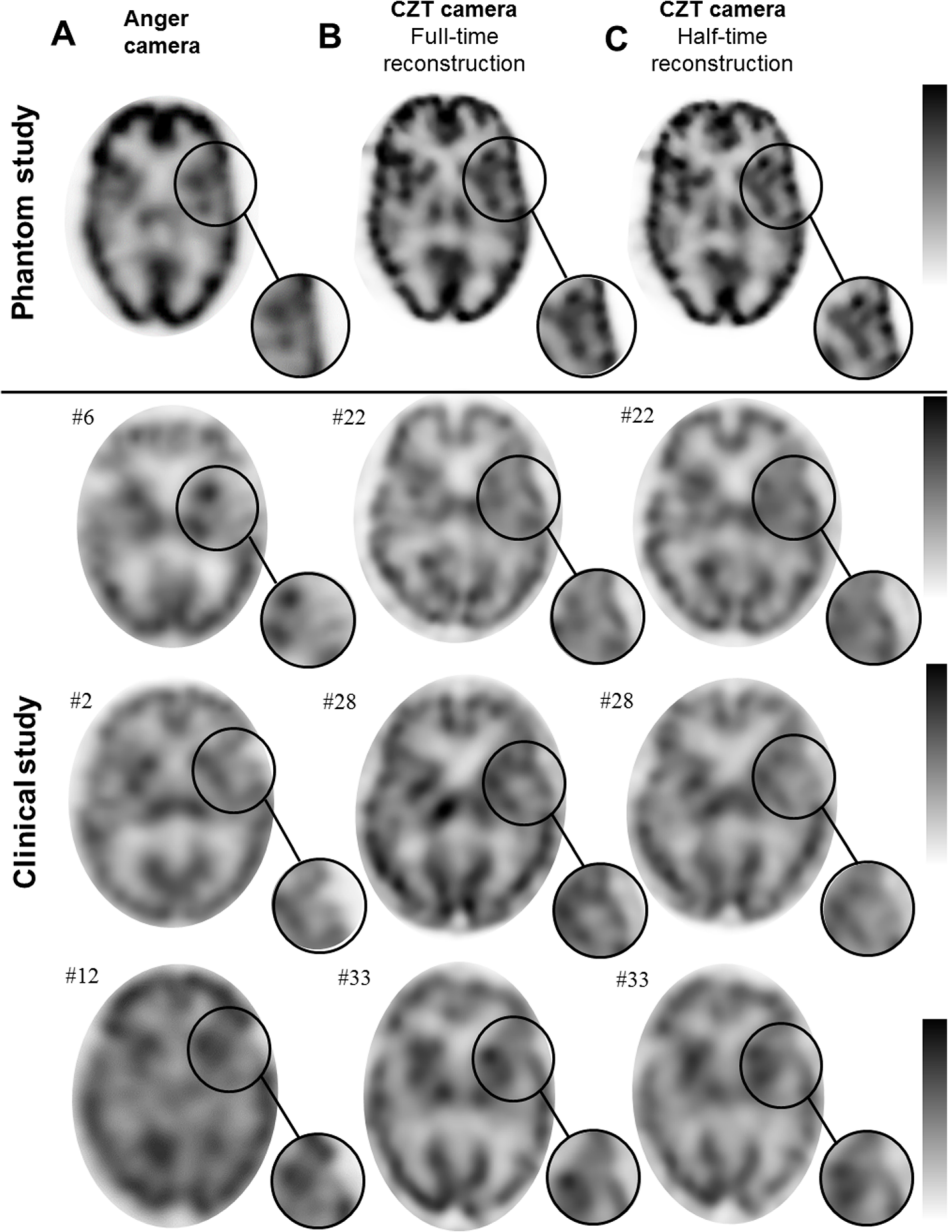
Representative examples of median axial brain ^99m^Tc-HMPAO SPECT slices obtained with the Anger camera (**a**) and with the CZT camera for full-time (i.e., 30 min, **b**) and half-time (i.e., 15 min, **c**) recordings, for the brain-phantom (upper slices) and for three matched pairs of patients imaged with the Anger or CZT camera

Representative examples of images from phantoms and patients obtained with the Anger camera and with full- and half-time recordings from the CZT camera are displayed on Fig. 3. Full sets of patient images obtained on both cameras are also available in a supplemental Figure 4.

## Discussion

The present study results, based on concordant data from phantom and patients, as well as from quantitative and qualitative visual analyses, show that the quality of brain perfusion SPECT images can be significantly enhanced by acquisition using a dedicated brain configuration for recordings obtained from a 360° CZT camera, as compared with an Anger camera. Furthermore, this enhancement may be achieved with a high-speed recording of no more than 15 min, a property that is particularly useful in patients with neurological diseases and for whom a brain perfusion SPECT may be indicated.

To date, CZT-SPECT studies published on brain perfusion [18, 20] or on dopamine receptors [32] are scarce and involve only very limited study populations. However, the superiority of CZT-based detectors over the NaI-scintillator systems utilized by current Anger cameras has already been clearly established, even in the clinical routine of SPECT investigations, although mostly for dedicated cardiac cameras [21, 33, 34]. In previous cardiac SPECT studies, dedicated cardiac CZT cameras were shown to provide significant enhancements in image quality and especially, in spatial resolution and image contrast [21]. However, these advantages are not only due to the CZT-based detectors, but also to the heart-centric focusing employed with these cardiac cameras which provides additional enhancements in count sensitivity and spatial resolution. Similar results are presented here for brain SPECT investigations performed with a CZT camera that is also capable of dealing with all current scintigraphy exams. Additionally, this 360° CZT camera provides an original geometry for acquisition. In particular, for head imaging, the 12 detectors surround the head and extend inwards to locate only a few centimeters from the head surface, i.e., in a configuration that could have been designed for a dedicated brain camera. This proximity of detectors is highly advantageous for spatial resolution and count sensitivity, and this advantage is further increased by a focal recording mode, enabling most of the recorded projections to be concentrated on a delineated brain area [20].

Results obtained on both phantom and patients give evidence of a more than 2-fold improvement of the tomographic count sensitivity of this CZT camera, as compared with that of a conventional 2-head Anger camera, in line with what has been previously reported in a case study report [20]. Image quality parameters were also significantly enhanced, as compared to the Anger camera for both phantom and patients (Fig. 2). It must be pointed out that the marked enhancement in the grey/white matter contrast was observed on CZT camera images despite the fact that the reconstruction process was optimized for contrast in a similar way for both cameras. In addition, although the enhancement in spatial resolution was documented here in an indirect way through a sharpness index at the grey/white matter interface, its magnitude is in line with what is observed for the full-width at half maximum (FWHM) of a punctual source placed at the central sites of fields-of-view (for central FWHM, CZTcamera 4.2 mm vs. Anger camera 4.8 mm, results not shown). By contrast, the relative noise was marginally higher on the images provided by the 360° CZT camera in comparison to those of the Anger camera, mainly due to the differences of the voxel dimensions between the two cameras (2.46 × 2.46 × 2.46 mm^3^ and 3.9 × 3.9 × 3.9 mm^3^ for CZT and Anger respectively). However, these noise level differences observed in phantoms were not significant in patients as is illustrated on the right lower panel of Fig. 2. Further results obtained with visual scoring give evidence that this enhancement in image quality is likely significant for the routine visual analysis of brain perfusion SPECT.

It should also be noted that for both cameras the sharpness index was lower, and thus, spatial resolution was higher for peripheral (occipital cortex) than for central (striatum) structures. This point could be subsequently confirmed by higher FWHM values for the punctual source positioned at approximately the same peripheral site (for tangential and radial FWHM respectively, CZT camera 3.0 mm and 3.5 mm vs. Anger camera 3.2 mm and 4.4 mm, results not shown). Such distance-related loss in spatial resolution is a common observation with the parallel hole collimator systems used with these cameras. It could be substantially lowered by further corrections, such as the application of a point spread function (PSF). However, such corrections are still mostly used in PET imaging, and at the time of this study, they were not available for our cameras.

A final observation was that the image quality provided by the CZT camera, as well as the magnitude of quality enhancement in comparison to the Anger camera, remained unchanged when only half of the recording times were used for the CZT camera image reconstruction (Fig. 2). This was achieved with a reduction in the number of iterations, thereby limiting the increase in image noise (Fig. 1). These enhancements, due to the combined improvements in count sensitivity and image quality provided by this CZT camera, give the opportunity to reduce injected activity (thus lowering patients’ radiation dose to less than 5 mSv) and/or recording time. The potential to limit recording time to only 15 min seems particularly advantageous for clinical routine in patients with cognitive or other neurological disorders (i.e., leading to enhanced comfort for the patient and a lower risk of motion artifacts). Such reduction in recording time, associated with a marked enhancement in image quality, could reposition brain perfusion SPECT to the forefront of brain imaging.

Today, brain perfusion SPECT is considered to be highly indicated (i) to image and localize the foci of epilepsy after injection of a perfusion tracer in the ictal state, whereas such ictal imaging is impossible with FDG PET [3]; (ii) to evaluate chronic cerebral ischemia with acetazolamide challenge, in order to detect areas of critically reduced perfusion [35]; and (iii) as an alternative to FDG PET for assessment of neurodegenerative disorders, particularly in diabetic patients for whom brain FDG-PET should be acquired in normal glycemia conditions [36, 37]. The new CZT cameras have also potential for new indications that may not be achievable with brain PET imaging. These later could involve multi-isotope acquisitions, similar to what is already planned for cardiac CZT-SPECT investigations [38, 39], due to the high energy resolution provided by CZT detectors. A possible example is the association of brain perfusion SPECT with DaT-SPECT in the exploration of Lewy-body diseases. In addition, with brain perfusion CZT-SPECT, brain activation studies could be planned for a very broad range of brain stimuli. Indeed, such studies are conventionally conducted with MRI but limited only to stimuli that are feasible for patients lying down within the magnet [40].

One limitation of the present study is that patient populations were different for each of the SPECT systems. Thus, patients imaged with the CZT camera had slightly lower MMSE scores than those imaged by the Anger camera. However, all other patient characteristics were similar between the two groups (Table 1), with all brain perfusion SPECT classified as normal after visual analysis and careful evaluation. Interestingly from this, a greater deterioration in image quality parameters would have been expected in the CZT camera group due to a higher level of brain disease in this group; however, this was not observed and therefore seemingly supports our observation of higher image quality provided by the CZT camera. Nevertheless, it is likely that further studies are required for confirmation in larger study populations, including patients with abnormal brain perfusion, as well as for determining whether the aforementioned improvements in image quality have a significant impact on diagnostic accuracy.

## Conclusion

The present study demonstrates that a much higher quality of brain perfusion images may be achieved with 360° CZT camera, as compared with a conventional Anger camera, even in high-speed recording conditions. It could therefore be speculated that these properties may lead to repositioning brain perfusion SPECT to the forefront of brain imaging.

## Supplementary Information

**Additional file 1: Supplemental Figure 4**. Image gallery of all axial brain ^99m^Tc HMPAO-SPECT slices acquired with the conventional Anger camera and the 360° CZT-camera with a full-time (i.e. 30 minutes; middle panel) and half-time (i.e. 15 minutes; lower panel) reconstruction, for matched pairs of patients imaged with the Anger- or CZT-cameras.

## References

[1] Kapucu ÖL, Nobili F, Varrone A, Booij J, Vander Borght T, Någren K (2009). EANM procedure guideline for brain perfusion SPECT using 99mTc-labelled radiopharmaceuticals, version 2. Eur J Nucl Med Mol Imaging..

[2] Quaranta D, Gainotti G, Di Giuda D, Vita MG, Cocciolillo F, Lacidogna G (2018). Predicting progression of amnesic MCI: the integration of episodic memory impairment with perfusion SPECT. Psychiatry Research: Neuroimaging..

[3] Verger A, Lagarde S, Maillard L, Bartolomei F, Guedj E (2018). Brain molecular imaging in pharmacoresistant focal epilepsy: current practice and perspectives. Revue Neurologique..

[4] Ritchie K, Gilham C, Ledésert B, Touchon J, Kotzki PO (1999). Depressive illness, depressive symptomatology and regional cerebral blood flow in elderly people with sub-clinical cognitive impairment. Age Ageing..

[5] Staffaroni AM, Elahi FM, McDermott D, Marton K, Karageorgiou E, Sacco S (2017). Neuroimaging in dementia. Semin Neurol..

[6] Tartaglia MC, Rosen HJ, Miller BL (2011). Neuroimaging in dementia. Neurotherapeutics..

[7] Joo EY, Seo DW, Hong S-C, Hong SB (2015). Functional neuroimaging findings in patients with lateral and mesio-lateral temporal lobe epilepsy; FDG-PET and ictal SPECT studies. J Neurol..

[8] Chandra PS, Vaghania G, Bal CS, Tripathi M, Kuruwale N, Arora A (2014). Role of concordance between ictal-subtracted SPECT and PET in predicting long-term outcomes after epilepsy surgery. Epilepsy Res..

[9] Bonte FJ, Harris TS, Hynan LS, Bigio EH, White CL (2006). Tc-99 m HMPAO SPECT in the differential diagnosis of the dementias with histopathologic confirmation. Clin Nuclear Med.

[10] Suzuki A, Takeuchi W, Ishitsu T, Morimoto Y, Kobashi K, Ueno Y (2015). High-resolution brain SPECT imaging by combination of parallel and tilted detector heads. Ann Nucl Med..

[11] Koulikov V, Lerman H, Kesler M, Even-Sapir E (2015). 99mTc-MDP bone scintigraphy of the hand: comparing the use of novel cadmium zinc telluride (CZT) and routine NaI(Tl) detectors. EJNMMI Res..

[12] Yamane T, Kondo A, Takahashi M, Miyazaki Y, Ehara T, Koga K (2019). Ultrafast bone scintigraphy scan for detecting bone metastasis using a CZT whole-body gamma camera. Eur J Nucl Med Mol Imaging..

[13] Silverman DHS (2004). Brain 18F-FDG PET in the diagnosis of neurodegenerative dementias: comparison with perfusion SPECT and with clinical evaluations lacking nuclear imaging. J Nucl Med..

[14] Farid K, Queneau M, Guernou M, Lussato D, Poullias X, Petras S (2012). First experience DaTSCAN imaging using cadmium-zinc-telluride gamma camera SPECT. Clin Nucl Med..

[15] Chikamori T, Goto K, Hida S, Miyagawa M, Ishimura H, Uchida K (2017). Diagnostic performance of a semiconductor gamma-camera system as studied by multicenter registry. J Cardiol..

[16] Melki S, Chawki MB, Marie PY, Imbert L, Verger A. Augmented planar bone scintigraphy obtained from a whole-body SPECT recording of less than 20 min with a high-sensitivity 360° CZT camera. Eur J Nucl Med Mol Imaging. 2020;47(5):1329–31. 10.1007/s00259-019-04525-y 31606830

[17] Imbert L (2018). Les caméras à semi-conducteurs grand champ : le point de vue du radiophysicien. Méd Nucléaire..

[18] Goshen E, Beilin L, Stern E, Kenig T, Goldkorn R, Ben-Haim S (2018). Feasibility study of a novel general purpose CZT-based digital SPECT camera: initial clinical results. EJNMMI Phys..

[19] Daou D (2019). Dedicated cardiac CZT SPECT is steadily moving to achieve its destiny. J Nucl Cardiol..

[20] Bordonne M, Marie P-Y, Imbert L, Verger A (2020). Brain perfusion SPECT acquired using a dedicated brain configuration on a 360° whole-body CZT-camera. J Neuroradiol..

[21] Imbert L, Poussier S, Franken PR, Songy B, Verger A, Morel O (2012). Compared performance of high-sensitivity cameras dedicated to myocardial perfusion SPECT: a comprehensive analysis of phantom and human images. J Nucl Med..

[22] Wu D, Zhang Z, Ma R, Guo F, Wang L, Fang W (2019). Comparison of CZT SPECT and conventional SPECT for assessment of contractile function, mechanical synchrony and myocardial scar in patients with heart failure. J Nucl Cardiol..

[23] Morelle M, Bellevre D, Hossein-Foucher C, Manrique A, Bailliez A. First comparison of performances between the new whole-body cadmium-zinc-telluride SPECT-CT camera and a dedicated cardiac CZT camera for myocardial perfusion imaging: Analysis of phantom and patients. J Nucl Cardiol. 2020;27(4):1261–9. 10.1007/s12350-019-01702-2 30963419

[24] Takahashi Y, Miyagawa M, Nishiyama Y, Ishimura H, Mochizuki T (2013). Performance of a semiconductor SPECT system: comparison with a conventional Anger-type SPECT instrument. Ann Nucl Med..

[25] Liga R, Gimelli A (2017). Imaging the heart’s brain: simultaneous innervation/perfusion analysis in the era of new CZT cameras. J Nucl Cardiol..

[26] Bienenstock EA, Ennis M (2014). The effect of object size on the sensitivity of single photon emission computed tomography: comparison of two CZT cardiac cameras and an Anger scintillation camera. EJNMMI Phys..

[27] Liu C-J, Cheng J-S, Chen Y-C, Huang Y-H, Yen R-F (2015). A performance comparison of novel cadmium-zinc-telluride camera and conventional SPECT/CT using anthropomorphic torso phantom and water bags to simulate soft tissue and breast attenuation. Ann Nucl Med..

[28] Niimi T, Nanasato M, Sugimoto M, Maeda H (2017). Evaluation of cadmium-zinc-telluride detector-based single-photon emission computed tomography for nuclear cardiology: a comparison with conventional anger single-photon emission computed tomography. Nucl Med Mol Imaging..

[29] American Psychiatric Association. Diagnostic and statistical manual of mental disorders (DSM-5®). American Psychiatric Pub. 2013.

[30] Nioche C, Orlhac F, Boughdad S, Reuzé S, Goya-Outi J, Robert C (2018). LIFEx: a freeware for radiomic feature calculation in multimodality imaging to accelerate advances in the characterization of tumor heterogeneity. Cancer Res..

[31] Salvadori J, Imbert L, Perrin M (2019). Head-to-head comparison of image quality between brain 18F-FDG images recorded with a fully digital versus a last-generation analog PET camera. EJNMMI Res..

[32] Bani Sadr A, Testart N, Tylski P, Scheiber C (2019). Reduced scan time in 123I-FP-CIT SPECT imaging using a large-field cadmium-zinc-telluride camera. Clin Nuclear Med.

[33] Lima R, Peclat T, Soares T, Ferreira C, Souza AC, Camargo G (2017). Comparison of the prognostic value of myocardial perfusion imaging using a CZT-SPECT camera with a conventional anger camera. J Nucl Cardiol..

[34] Salvadori J, Petegnief Y, Sabbah R, Morel O, Boulahdour H, Karcher G (2019). Compared vulnerabilities to small cardiac motions between different cameras used for myocardial perfusion imaging. J Nucl Cardiol..

[35] Vagal AS, Leach JL, Fernandez-Ulloa M, Zuccarello M (2009). The acetazolamide challenge: techniques and applications in the evaluation of chronic cerebral ischemia. AJNR Am J Neuroradiol..

[36] Varrone A, Asenbaum S, Vander Borght T, Booij J, Nobili F, Någren K (2009). EANM procedure guidelines for PET brain imaging using [18F]FDG, version 2. Eur J Nucl Med Mol Imaging..

[37] Waxman AD, Herholz K, Lewis DH, Herscovitch P, Minoshima S, Ichise M, Drzezga AE, Devous MD, Mountz JM. Society of Nuclear Medicine Procedure Guideline for FDG PET Brain Imaging Version 1.0, approved Feb 8, 2009.

[38] Wagenaar DJ, Parnham K, Sundal B, Maehlum G, Chowdhury S, Meier D, et al. Advantages of semiconductor CZT for medical imaging. SPIE Proceedings Vol. 67079, Penetrating Radiation Systems and Applications VIII, 67070I. 2007.

[39] Makita A, Matsumoto N, Suzuki Y, Hori Y, Kuronuma K, Yoda S (2016). Clinical feasibility of simultaneous acquisition Rest ^99m^Tc/Stress ^201^Tl dual-isotope myocardial perfusion single-photon emission computed tomography with semiconductor camera. Circ J..

[40] Verger A, Guedj E (2018). The renaissance of functional 18F-FDG PET brain activation imaging. European Journal of Nuclear Medicine and Molecular Imaging..

