# Supplementary figures and images for: High-quality brain perfusion SPECT images may be achieved with a high-speed recording using 360° CZT camera
Source: EJNMMI Phys. 2020 Nov 4;7:65. doi: 10.1186/s40658-020-00334-7 (PMC7642149; doi:10.1186/s40658-020-00334-7)

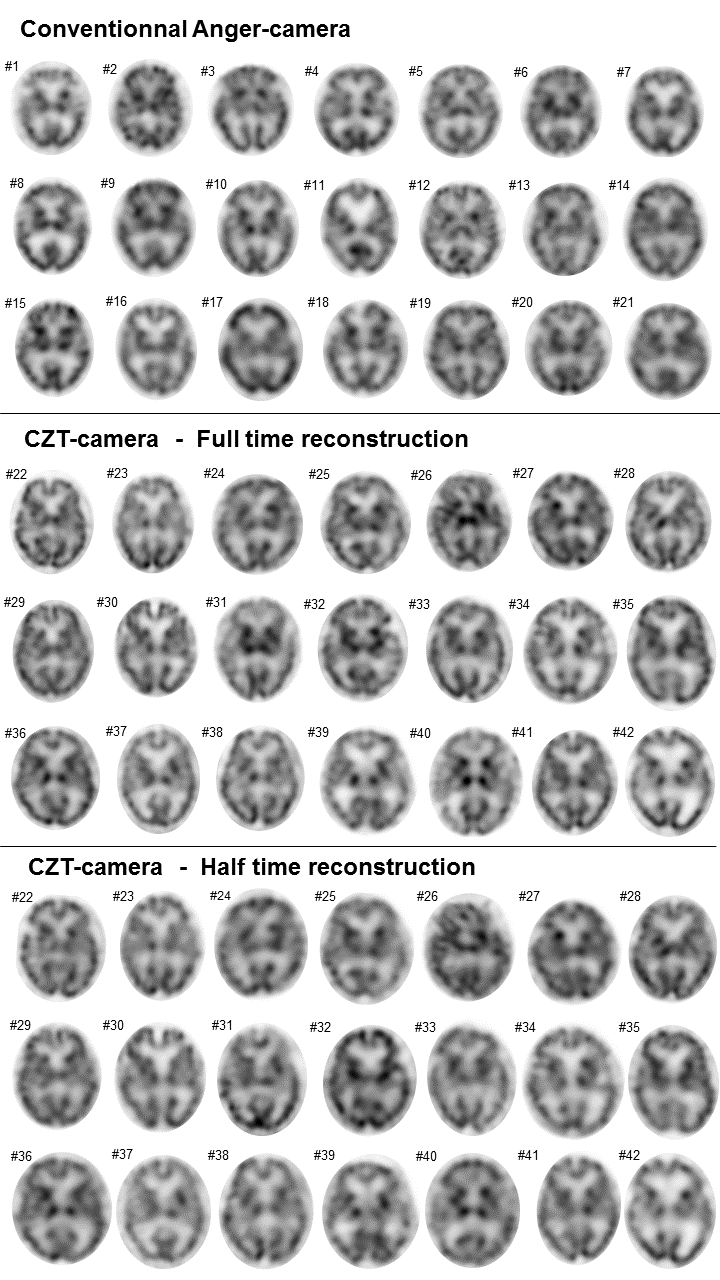

Supplement: Supplementary file 1 — Additional file 1: Supplemental Figure 4. Image gallery of all axial brain 99mTc HMPAO-SPECT slices acquired with the conventional Anger camera and the 360° CZT-camera with a full-time (i.e. 30 minutes; middle panel) and half-time (i.e. 15 minutes; lower panel) reconstruction, for matched pairs of patients imaged with the Anger- or CZT-cameras. [file 40658_2020_334_MOESM1_ESM.png]
